# Transcriptional and Post-transcriptional Control of the Nitrate Respiration in Bacteria

**DOI:** 10.3389/fmolb.2021.667758

**Published:** 2021-05-07

**Authors:** Sylvain Durand, Maude Guillier

**Affiliations:** CNRS, UMR 8261, Université de Paris, Institut de Biologie Physico-Chimique, Paris, France

**Keywords:** denitrification, dissimilatory nitrate reduction, two-component systems, FNR, nitrate reductase, small RNAs, Hfq

## Abstract

In oxygen (O_2_) limiting environments, numerous aerobic bacteria have the ability to shift from aerobic to anaerobic respiration to release energy. This process requires alternative electron acceptor to replace O_2_ such as nitrate (NO_3_^–^), which has the next best reduction potential after O_2_. Depending on the organism, nitrate respiration involves different enzymes to convert NO_3_^–^ to ammonium (NH_4_^+^) or dinitrogen (N_2_). The expression of these enzymes is tightly controlled by transcription factors (TFs). More recently, bacterial small regulatory RNAs (sRNAs), which are important regulators of the rapid adaptation of microorganisms to extremely diverse environments, have also been shown to control the expression of genes encoding enzymes or TFs related to nitrate respiration. In turn, these TFs control the synthesis of multiple sRNAs. These results suggest that sRNAs play a central role in the control of these metabolic pathways. Here we review the complex interplay between the transcriptional and the post-transcriptional regulators to efficiently control the respiration on nitrate.

## Introduction

**FIGURE 1:**
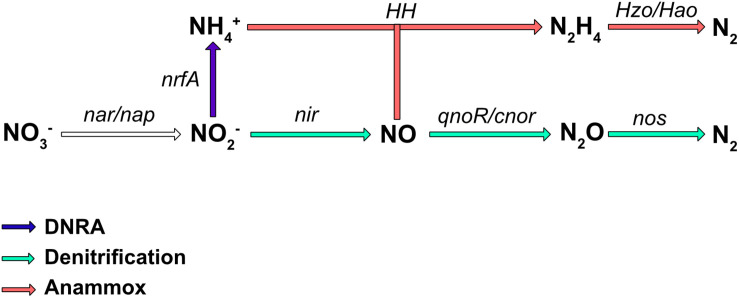
Biological pathways of nitrate respiration in bacteria. The major process involved in nitrate transformation to ammonium (NH_4_^+^) is the DNRA pathway (in blue), while those involved in nitrate transformation to N_2_ are denitrification (green), and ammonium oxidation (anammox; red). The main enzymes involved are Nar, a membrane-bound dissimilatory nitrate reductase; Nap, a periplasmic dissimilatory nitrate reductase; NirBD, a nitrite reductase; NrfA and NirK/NirS, nitrite reductases; cNor and qNor, nitric oxide reductases; NosZ, a nitrous oxide reductase; HH, hydrazine hydrolase; HAO, hydrazine oxidoreductase.

Nitrate is an important nutrient for microorganisms and can be used in both assimilatory and dissimilatory pathways. Assimilatory pathways allow the incorporation of nitrogen into the organism’s biomass (DNA, proteins.). In contrast, dissimilatory nitrate reduction to ammonium (DNRA) is more a catabolic pathway leading to nitrogen excretion. In some cases, the dissimilatory process can release energy and is therefore termed “nitrate respiration.” Several metabolic pathways, such as DNRA, denitrification, and in, some bacteria, anaerobic ammonium oxidation (anammox) (reviewed in Kartal et al., 2012), use the reduction of nitrate or nitrite as the first step in respiration. The final products are NH_4_^+^ for DNRA, and N_2_ for the denitrification and anaerobic anammox pathways (Figure 1); however, numerous bacteria are only partial denitrifiers since the last enzyme required to convert N_2_O to N_2_ is absent (Philippot et al., 2011). These diverse respiratory nitrate reduction pathways are not necessarily present simultaneously in a given organism, but can compete for nitrate utilization. Denitrification is probably the best studied nitrate pathway in bacteria and it has been shown that incomplete denitrification can release N_2_O in the atmosphere, a greenhouse gas involved in global warming. Nitrate reduction also plays a key role in bacterial pathogenesis (Vázquez-Torres and Bäumler, 2016) as well as in the gut colonization by enterobacteria.

Numerous enzymes are involved in these different metabolic pathways, and the coordination of their expression is absolutely required to limit the energetic cost and avoid the accumulation of toxic compounds such as nitric oxide (NO), a free radical which has the propensity to react with other molecules (see below). To correctly regulate the expression of these genes, bacteria use several transcription factors (TFs). One of the most prominent families of transcriptional regulators found to control nitrate respiration is that of the FNR-like proteins.

As it has been shown for other important metabolic pathways, transcriptional regulation is often combined with post-transcriptional control, where non-coding small RNAs (sRNAs) play a key role. Several studies have highlighted the role of sRNAs in nitrogen metabolism and this aspect was reviewed recently (Prasse and Schmitz, 2018; Muro-Pastor and Hess, 2020). However, recent developments also pointed to an important role for sRNAs in nitrate respiration pathways, which will be addressed in this review.

We will briefly present the diversity of enzymes involved in these metabolic pathways and how the expression of these enzymes is regulated by TFs and sRNAs.

## The Diversity of Enzymes Involved in Nitrate Respiration

### Nitrate Reductase (Nar, Nap)

**FIGURE 2:**
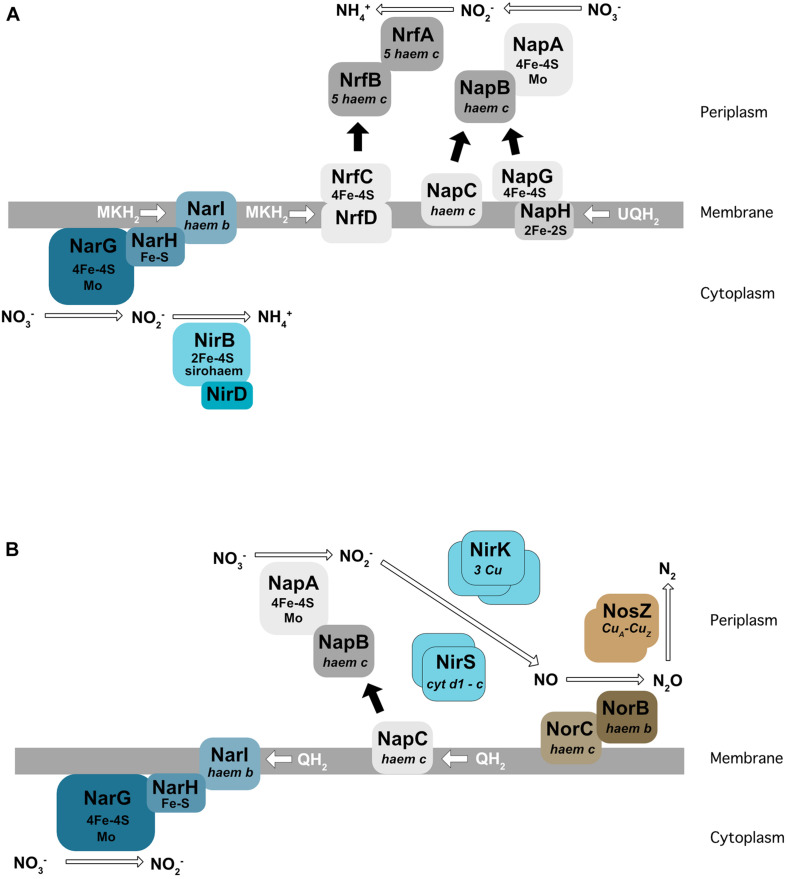
Enzymes involved in dissimilatory nitrate reduction to ammonium (DNRA) in *E. coli* **(A)** and in denitrification in *P. denitrificans* **(B)**. Nar, membrane-bound dissimilatory nitrate reductase; Nap, periplasmic dissimilatory nitrate reductase; NirBD, a sirohaem containing nitrite reductase; NrfA, a cytochrome c nitrite reductase; NirK/NirS, cNor, and qNor, Cu-containing/cd1-type nitric oxide reductases; NosZ, a nitrous oxide reductase; QH2, MKH2, UQH2 : quinone, menaquinone, ubiquinone.

For all nitrate respiration pathways mentioned above, the first step consists of the conversion of nitrate to nitrite (NO_2_^–^) by a nitrate reductase. These enzymes use molybdenum as a co-factor. Two types of nitrate reductase have been shown to be involved in nitrate respiration: Nar localized at the membrane and Nap found in the periplasm (Figure 2). The Nar nitrate reductases are trimeric (NarG/H/I) and encoded by the *NarGHJI* operon. This operon is widely conserved in bacteria and is found in nitrate-ammonifying (DNRA) bacteria from *Escherichia coli* to *Bacillus subtilis*. NarI is a transmembrane protein that receives electrons from the quinone pool and anchors NarG and NarH at the membrane. The electrons are transferred from NarI, a cytochrome *b* quinol oxidase, to NarG *via* NarH. NarH and NarG contain Fe-S clusters that receive an electron and transfer it to the active site of NarG (Figure 2A). The Nar reductase couples nitrate respiration to proton translocation across the membrane, driving ATP generation. NarJ, encoded by the last gene of the operon, is a chaperone protein that plays a role in the maturation and membrane insertion of Nar. Interestingly, *E. coli* and *Salmonella typhimurium* possess a duplication of this operon (*narZYWV*), which seems to play a greater role in stressed cells rather than anaerobic respiration (Blasco et al., 1990; Spector et al., 1999). Three nitrate reductases (Nar1, Nar2, and Nar3) have been identified and characterized in *Streptomyces coelicolor*. Nar1 is induced in spores and competes with aerobic respiration for electrons from menaquinol (Falke et al., 2019). In contrast, Nar2 and Nar3 are induced by anoxic condition in the mycelium (Urem et al., 2016; Fischer et al., 2019).

The Nap nitrate reductases are found in the periplasm of many different Gram-negative bacteria (reviewed in Richardson et al., 2007). The NapA subunit, bearing the catalytic active site, is transported across the membrane by the Tat apparatus and requires the NapD chaperone encoded in the same operon (Grahl et al., 2012). NapA receives electrons from two cytochrome *c* proteins, the NapC membrane protein and the NapB subunit (review by Richardson et al., 2001) (Figure 2B). In *E. coli*, the *nap* operon also encodes for a quinol-oxidizing system (*napGH*) composed of two Fe-S proteins: (1) NapH, a membrane-bound quinol dehydrogenase and (2) NapG, a periplasmic electron transfer adapter protein which can, similarly to NapC, deliver electrons to NapB. However, the exact function of this complex in *E. coli* is still unclear. The presence of both NapC and NapGH proteins in bacterial genomes is variable. Some bacteria such as *Pseudomonas aeruginosa* only have the *napC* gene and others, e.g., ε-proteobacteria such as *Campylobacter jejuni* or *Sulfurimonas denitrificans*, only have NapGH (Sievert et al., 2008; Kern and Simon, 2009; reviewed in Sparacino-Watkins et al., 2014). The *nap* operon also encodes NapF in *E. coli*, which is proposed to post-translationally modify NapA prior to its export into the periplasm (Nilavongse et al., 2006). Interestingly, this protein is absent in its close relative *Salmonella*. In contrast to the Nar, Nap enzymes are unable themselves to generate a proton motive force. However, Nap can play a role in nitrate respiration when associated with a proton translocating enzyme such as the NADH dehydrogenase NuoA-N (Moreno-Vivián et al., 1999). In this way, NapA has been shown to support anaerobic respiration in *E. coli* (Stewart et al., 2002), and in several bacteria like *Bradyrhizobium japonicum* (Bedmar et al., 2005), *Rhodobacter sphaeroides* (Liu et al., 1999), *Pseudomonas* sp. strain G-179 (Bedzyk et al., 1999), where the Nap enzyme has been shown to be used for the first step of denitrification rather than Nar.

### Nitrite Reductase (Nir, Nrf)

The nitrite reductases ensure the reduction of nitrite to NO in denitrifying bacteria (denitrification) and into ammonium in DNRA bacteria, also known as ammonifiers (see below).

In denitrifying bacteria, two types of unrelated nitrite reductases (Nir) have been identified so far (reviewed in Moura and Moura, 2001). One, namely NirK, is an homotrimeric copper-containing enzyme which has been found in both Gram-negative and -positive bacteria (Suharti and de Vries, 2005). The second, NirS, is a homodimeric cytochrome *cd1* nitrite reductase that has been characterized in Gram-negatives (Figure 2B). Electrons are delivered by the cytochrome *bc1* complex. The *nirS* gene cluster encodes numerous additional proteins (for example the *nirXISECFDLGHJN* operon in *Paracoccus denitrificans*) necessary for the correct assembly of the active site haem *d1* cofactor of NirS (reviewed in Bali et al., 2014). In contrast to *nirS*, the *nirK* gene is generally isolated, but in some cases is in an operon with the *nirV* gene, whose product may be required for the correct insertion of the copper center. The presence of NirK and NirS has been for a long time proposed to be mutually exclusive. However, recent genomic comparisons have showed that both enzymes can be present in the same organism (Graf et al., 2014), probably with different roles and regulation, notably in *Pseudomonas stutzeri* (Wittorf et al., 2018).

In the nitrate respiratory ammonifiers, such as *E. coli* and *Salmonella enterica*, NirK and NirS as well as the nitric oxide reductases (qNor and cNOR, see below) are absent, and the reduction of nitrite is performed by two classes of enzymes:

(1) A cytoplasmic sirohaem-containing nitrite reductase (NirBD), encoded by *nirB* and *nirD* (Figure 2A), with the *nirC* gene in the same operon probably encoding a nitrite transporter. The *nirB* and *nirD* genes are also present in the genome of *B. subtilis* (*nasDEF*). The *nasDE* genes encode the nitrite reductase subunits and *nasF* is required for nitrite reductase sirohaem cofactor formation. Interestingly, this enzyme is used for both anaerobic respiration and nitrogen assimilation in *B. subtilis* (Ogawa et al., 1995; Nakano et al., 1998).
(2) The periplasmic Nrf enzyme, consisting of a pentahaem cytochrome *c* nitrite reductase that reduces nitrite by using formate as an electron donor (Einsle et al., 2002; Figure 2A). NrfA was first identified in *E. coli*, but is also present in the periplasm of numerous γ-, δ-, and ε-proteobacteria. Similar to the nitrate reductase described previously, electrons are transferred from the membrane quinol dehydrogenase (NrfH or NrfCD) to the cytochrome *c* reductase NrfA via the small electron transfer protein NrfB (reviewed by Simon, 2002). In *E. coli*, the *nrf* operon also encodes three proteins (NrfEFG) dedicated to the attachment of the active site haem group.

Interestingly, NO is also produced by *E. coli* and *S. enterica*, despite the fact that they do not possess NirK or NirS enzymes, and is proposed to be a side-product of nitrate respiration. It has been shown that nitrite reduction to NO can be done by the NarG enzyme in *Salmonella* and represents the major route of NO production in this organism (Gilberthorpe and Poole, 2008).

### Nitric Oxide Reductase (qNOR, cNOR)

The nitric oxide reductases reduce NO into nitrous oxide (N_2_O). They are part of the heme-copper oxidase family but are not able to translocate protons, in contrast to the other enzymes of this family (Bell et al., 1992; Hino et al., 2010; reviewed in Garcia-Horsman et al., 1994). Two types of NOR have been identified in Gram-negative bacteria: cNOR and qNOR, depending on the electron donor (heme *c* in cNOR; menaquinol in qNOR). cNOR is composed of two subunits: NorB with the catalytic site receiving electrons from a periplasmic electron donor *via* the NorC membrane bound cytochrome *c* (Figure 2B). cNORs are only found in denitrifying bacteria. Interestingly, a transient interaction between the cNORs and NirS (the *cd1* nitrite reductase) has been proposed in *P. denitrificans* (Albertsson et al., 2019), and a similar interaction was observed in *P. aeruginosa*. This interaction is thought to be important for the channeling of the toxic intermediate NO. The NorCB structural subunits are encoded in an operon with other genes (*norEFCBQD*). The *norQ* and *norD* gene are always linked to *norCB*, but the *norE* and *norF* genes can be encoded separately or even be absent (Zumft, 2006). The function of these accessory proteins is not well defined. A structural study showed that even if cNOR and qNOR enzymes are not able to pump protons, the catalytic protons can be transferred from bulk water to the buried active site through a specific pathway consisting of a water channel and/or a hydrogen-bond network (Shiro, 2012).

In contrast to cNOR, the qNOR enzyme is a unique single subunit protein (NorZ) that receives electrons from quinones. The qNOR enzyme has some sequence similarities with both the NorB and the NorC proteins (Shiro, 2012). The qNOR enzymes have a wider taxonomic distribution and are found in some *Archaea* and non-denitrifying pathogenic bacteria, where they play a role in NO detoxification (Hendriks et al., 2000; Sousa et al., 2012).

In the Gram-positive *Bacillus azotoformans*, a third type of NOR was identified that binds copper (Cu_A_NOR). This enzyme receives electrons from membrane-bound cytochrome C_551_ (Al-Attar and De Vries, 2015) and the large subunit is similar to NorB. The small subunit has no heme *c*, but a Cu_A_ binding site.

Other non-respiratory enzymes have been identified that can detoxify NO. For example the flavohaemoglobin Hmp which is widespread in bacteria converts NO to NO_3_^–^ (Vinogradov et al., 2013), and the flavorubredoxin NorV, with its NADH dependent oxidoreductase NorW (Gomes et al., 2002), reduces NO to NO^–^. Hmp and NorV are both present in *E. coli* but their mutation does not impair the reduction of NO, meaning that other NO reduction pathways still need to be characterized (Vine and Cole, 2011). Since these enzymes are not considered to play a role in respiration, we will not discuss them further in this review.

### Nitrous Oxide Reductase (Nos)

Nitrous oxide reductase (Nos) is the last enzyme to complete denitrification by reducing N_2_O into dinitrogen (N_2_). In most denitrifying bacteria, this is a homodimeric copper-enzyme (NosZ) localized in the periplasm (Figure 2B). However, this enzyme is membrane bound in the Gram-positive bacteria *B. azotoformans* and *Thiobacillus denitrificans* (Hole et al., 1996; Suharti and de Vries, 2005). It was shown in several bacteria, such as *P. denitrificans*, that electron transfer to Nos is from cytochrome *c*. NosZ is in general found in an operon with at least 5 other genes (*nosRZDFYL*), encoding proteins required for its expression, maturation and maintenance (Zumft, 2006). This operon sometimes encodes supplementary genes, such as *nosX* or *nosC*. In α-, β-, and γ-proteobacteria, the NosR and NosX proteins can bind flavin mononucleotide (FMN) that might be an alternative transport pathway from the quinone pool to NosZ. The NosDFY and L proteins are involved in the maturation of NosZ, and the function of NosC is unknown.

Numerous genomes of ammonifiers also encode an atypical nitrous oxide reductase (cNosZ) (Sanford et al., 2012; Jones et al., 2013). cNosZ is a variant of the canonical NosZ enzyme, but fused to a cytochrome *c* domain which transports electrons to the active site (Simon et al., 2004). Moreover, cNosZ is exported to the periplasm by the Sec secretion pathway and not by the Tat (Twin Arginine translocation) pathway like the classical NosZ enzyme. The genome of *Wolinella succinogenes* encodes this atypical enzyme in a large operon, *nosZBDGC1C2HFYL*. The NosG and H are menaquinol dehydrogenases, homologous to the NapGH proteins. The 2 cytochrome *c* proteins (NosC1 and C2) form a putative electron transport pathway from menaquinol to cNosZ. The *nosDFY* genes encode a membrane-bound ABC transporter that might be important for the maturation of atypical Nos systems. Finally, NosL could be a chaperone involved in copper metallocenter assembly (Zumft, 2006).

For a long time, the denitrification and the nitrate ammonification pathways were considered to be mutually exclusive. However, recent genome sequencing data have revealed that some bacteria have all the genes required for both pathways. *Shewanella loihica*, for example, encodes the NrfA enzyme for the DNRA pathway, and NirK, NorB and NosZ enzymes for the denitrification (Sanford et al., 2012). Both pathways are functional in this bacterium (Yoon et al., 2015) and depend on the C/N ratio. Indeed, at low C/N ratio (i.e., electron donor-limiting growth condition) the denitrification pathway is preferred to DNRA. In contrast, at high C/N ratio (i.e., electron-acceptor limiting growth condition), ammonification is dominant (Yoon et al., 2015). Temperature and pH also influence the nitrate pathways, showing that nitrate respiration is widely connected to environmental conditions.

Some bacteria are also able to release energy by forming N_2_ gas from ammonium and nitrite under anoxic conditions (Figure 1). This energy pathway (anammox) seems to be limited to the *Planctomycetales* order. These bacteria have been found in almost all aquatic habitats where oxygen is limiting. This specific pathway requires two type of enzymes, hydrazine hydrolase (HH), which produces hydrazine from ammonium and NO, and hydrazine oxidoreductase (HZO/HAO), which allows the oxidation of hydrazine to N_2_ (Figure 1). Electrons are thought to be transferred to the quinone pool to ultimately generate a proton motive force.

The numerous enzymes involved in nitrate utilization pathways highlight the need for bacteria to efficiently control their expression at the transcriptional and post-transcriptional levels in response to their specific environments. This control is discussed below, with a focus on the few bacterial species where this has been studied in most detail: *E. coli*, *S. enterica*, and *B. subtilis* for the DNRA pathway, and *P. denitrificans* and *P. aeruginosa* for denitrification.

## Transcriptional Regulators Controlling the Synthesis of Enzymes Involved in Nitrate Respiration

In bacteria that have the ability to respire on nitrate, this mode of respiration mainly depends on three parameters:

(1) Oxygen limitation
(2) The presence of nitrate (NO_3_^–^) or derived compounds such as nitrite (NO_2_^–^) or nitric oxide (NO) in the medium
(3) An appropriate donor of electrons (i.e., organic carbon compounds).

Several transcriptional regulators play a key role in integrating these signals to modulate the expression of genes related to nitrate respiration. Numerous studies have highlighted the importance of both the FNR-like global TFs controlling several metabolic pathways in order to prepare the cell to anaerobic conditions and the more specific two-component systems (TCSs) that frequently control one metabolic pathway. Most of these regulators can sense different signals like O_2_, NO_3_^–^, NO_2_^–^ or NO (see below). FNR uses an O_2_-labile [4Fe-4S]^2+^ cluster to sense oxygen. In the absence of O_2_, the cluster is stable and forms a homodimer able to bind its DNA target sequences. In presence of O_2_, the [4Fe-4S]^2+^ cluster is unstable and the protein cannot dimerize, rendering it inefficient at binding its targets. It has also been demonstrated that the [4Fe-4S]^2+^ cluster is sensitive to NO *in vitro*, which also reduces the DNA-binding ability of FNR (Crack et al., 2013). Depending on the position of the FNR binding site in the promoter region, FNR activates or represses gene expression. In *E. coli* and *B. subtilis*, the FNR regulon represents more than 100 genes and its main role is to prepare the cell for anaerobic respiration (Constantinidou et al., 2006; Reents et al., 2006).

Two-component systems are typically composed of a membrane-bound histidine kinase that senses a specific stimulus and a response regulator that mediates the cellular response by modifying target gene expression. Numerous TCSs, such as NarX-NarL, are involved in nitrate respiration and will be described below. In general, they are more dedicated to nitrate respiration than FNR and their regulon includes less genes than the FNR-like regulon.

This strategy to use both global and more specific transcriptional regulators is similar in numerous bacteria, using either the DNRA or the denitrification pathway to respire on nitrate. This can provide significant advantages that include the integration of several environmental cues, e.g., O_2_ availability via FNR or FNR-like global regulators and the presence of alternative electron acceptors such as nitrate, via the NarX-NarL and NarQ-NarP TCSs. Some variations on this theme exist as well. For example, in *B. subtilis* and *Streptomyces* (see below), no transcriptional regulator that directly senses nitrate has been described, i.e., the regulation of the genes involved in the respiration on nitrate is not directly influenced by the presence of nitrate in the environment in this organism.

The different transcriptional regulators, the signals to which they respond, and their targets linked to nitrate respiration are described in the following sections.

### Transcriptional Regulation of the DNRA Pathway

**FIGURE 3:**
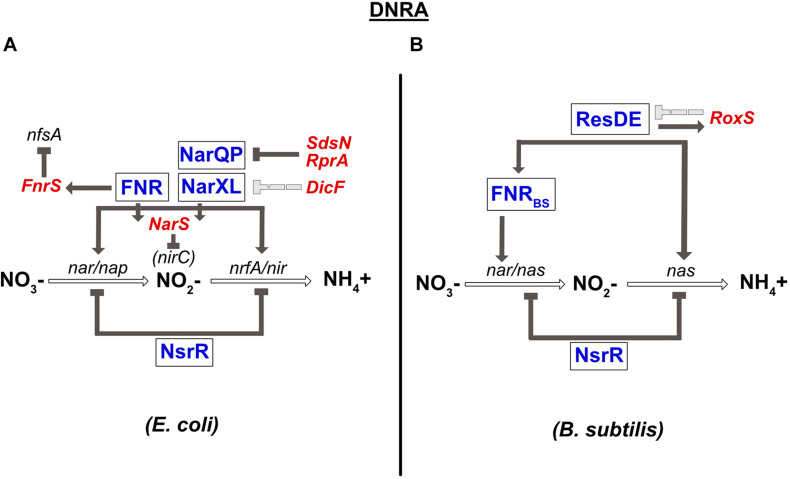
Regulation of dissimilatory nitrate reduction to ammonium (DNRA) in *E. coli* **(A)** and *B. subtilis* **(B)**. **(A)** Nitrate reductase (*nar/nap*) and nitrite reductase (*nir/nrfA*) genes are co-regulated by FNR and by the two-component system NarQP for the *nap*, *nir*, and *nrfA* operons and NarXL for the *nar* and *nir* genes. The *nar* and *nir* operons are also repressed by the NsrR TF. *narP* expression is inhibited by two sRNAs, SdsN, and RprA. Expression of *narXL* is repressed, probably indirectly, by the DicF sRNA. In contrast, the FNR TF is responsible for *fnrS* and *narS* expression. FnrS regulates numerous genes that are dispensable during anaerobic respiration, such as *nfsA*, encoding an NADPH-dependent nitroreductase. The NarS sRNA is encoded at the 3′ end of the *narK* mRNA (encoding a transporter of nitrate and nitrite) and represses the expression of *nirC*, coding for a nitrite transporter. **(B)** Nitrate reductase gene (*nar*) is regulated by FNR. FNR is itself under the control of the ResDE TCS and also controls the expression of nitrite reductase (*nas*). NsrR inhibits the expression of the *nas* operon coding for nitrate and nitrite reductase. ResDE is a transcriptional activator of expression of the RoxS sRNA, which in turn negatively impacts its expression. RoxS regulates numerous genes encoding enzymes of the TCA cycle.

A complete review on the conservation of the transcriptional regulators involved in DNRA has been published previously (Rodionov et al., 2005). Briefly, the global transcriptional regulator FNR sensing O_2_, and the two nitrate-sensing NarQ-NarP and NarX-NarL TCSs have been shown to play a central role in the regulation of anaerobic respiration in *E. coli* and numerous γ-proteobacteria. In the presence of nitrate, NarX and NarQ phosphorylate NarL and NarP, respectively. Importantly, cross-regulation exists between these two TCSs; NarQ additionally efficiently controls the phosphorylation status of NarL, while NarX can influence NarP much more poorly (Noriega et al., 2010). Another difference between these two sensors is that NarQ is sensitive to signals other than nitrate, especially nitrite, but not NarX (Lee et al., 1999). All these transcriptional regulators (FNR, NarXL, and NarQP) are involved in controlling the synthesis of enzymes of the nitrate reduction pathway (Figure 3). More precisely, they all activate the expression of the *nirBDC* operon encoding nitrite reductase. The *narGHJI* operon is activated by NarL and FNR. *E. coli* and *S. typhimurium* genomes also encode a second cytoplasmic nitrate reductase (*narZYWV*), whose role is still unclear. However, in contrast to the *narGHJI* operon, these genes are repressed by FNR in *S. typhimurium* (Spector et al., 1999). The *nap* and the *nrf* genes encoding for the periplasmic nitrate- and nitrite reductase, respectively, are controlled by the NarP response regulator and by FNR (Figure 3; Jayaraman et al., 1987; Lamberg and Kiley, 2000; Browning et al., 2004). Several other targets of NarX-NarL and NarQ-NarP encode proteins involved in pathways related to nitrate respiration, e.g., NO detoxification (Filenko et al., 2005) or molybdenum attachment to enzymes such as nitrate reductase (Hasona et al., 2001). Another important transcriptional regulator involved in nitrate respiration in *E. coli* is NsrR. This transcriptional repressor has an NO-sensitive [Fe-S] cluster and is not only present in β- and γ-proteobacteria, but also in *Streptomyces* and *Bacillus* species (Rodionov et al., 2005; reviewed in Tucker et al., 2010). Nitrosylation of this factor inhibits its DNA-binding activity, leading to the derepression of the genes controlled by NsrR. In *E. coli*, NsrR has been shown to regulate 9 operons, including the nitrate- and nitrite reductase operons (*nap* and *nrf*) (Filenko et al., 2007; Figure 3). In contrast, the *nrf* operon is not sensitive to the mutation of *nsrR* in *S. typhimurium* (Browning et al., 2010). NsrR was also shown to regulate expression of the Nir and Nor enzymes in denitrifying bacteria such as *Neisseria meningitidis* (Heurlier et al., 2008). At last, it should be highlighted that enzymes of the nitrate respiration pathways are most often metalloenzymes that require redox-active cofactors, such as molybdenum, iron or copper (reviewed in Garcia-Horsman et al., 1994, Moreno-Vivián et al., 1999, Sullivan et al., 2013). Interestingly, two regulators of the *E. coli nap* operon are themselves dependent on such cofactors: ModE, whose activity is induced under anaerobic conditions through molybdenum binding, activates *nap* expression (McNicholas and Gunsalus, 2002) while IscR, containing a 2Fe-2S cluster, represses it under aerobic conditions (Giel et al., 2006). ModE is able to override the control of *napF* by nitrate when molybdenum is limiting (McNicholas and Gunsalus, 2002).

In *Mycobacteria*, NarL is also responsible for the activation of the *narGHJI* operon. Although this protein has 74% identity to *E. coli* NarL, the sensor kinase NarS is not related to NarX. Another TCS, namely DevR/DevS, was identified in *Mycobacteria* and responds to anaerobic conditions. DevR, the response regulator, also controls the expression of the *nar* genes and the nitrate transporter NarK2. Interestingly and in contrast to what is known in α- and γ-proteobacteria, the two response regulators DevR and NarL were proposed to form a heterodimer that can efficiently bind the *narK2* promoter in *Mycobacterium tuberculosis* (Malhotra et al., 2015). Indeed, *in vitro* binding assays showed that DevR binding on *narK2* promoter is stimulated in presence of NarL. More recently, it was shown that the PrrAB TCS, also found in α-proteobacteria (see below), induced the transcription of the DevR response regulator in *Mycobacterium smegmatis* and is important for ATP generation under aerobic and hypoxic conditions (Maarsingh et al., 2019).

The NarX-NarL TCS is functionally replaced by the NreABC system in *Staphylococci*. NreABC has the ability to sense both O_2_ and nitrate, with a higher impact of oxygen compared to nitrate. NreA has a nitrate-binding GAF domain that senses nitrate and NreB, considered as a functional equivalent to FNR, has a [4Fe-4S]^2+^ cluster that is sensitive to O_2_ (Müllner et al., 2008). NreB is the sensor histidine kinase and NreC the response regulator of the NreBC TCS. NreB is auto-phosphorylated in anoxic conditions, leading to the phosphorylation of NreC (NreC-P). NreC-P was shown to bind to the GC rich palindromic region of the *narGHIJ*, *nir* and *narT* genes (Schlag et al., 2008). The NreA protein represents an additional layer of regulation for this TCS. Indeed, in the absence of nitrate, NreA interacts with NreB and inhibits NreB kinase activity (Niemann et al., 2014; Nilkens et al., 2014).

In *B. subtilis*, the *narGHJI* operon is regulated by FNR_BS_ (Figure 3). In contrast to *E. coli* FNR, the [4Fe-4S]^2+^ cluster of FNR_BS_ is at the C-terminus of the protein (Reents et al., 2006). Interestingly, in this bacterium, nitrate has no direct effect on the *narGHJI* operon, which seems to be regulated by FNR alone. Indeed, nitrate sensors such as NarXL and NreA have not been identified in *B. subtilis*. However, on one hand, FNR is transcriptionally regulated by the ResDE TCS that responds to both O_2_ and NO (Nakano et al., 1996; Nakano and Zhu, 2001; Geng et al., 2007), and on the other, the *fnr* gene is co-transcribed with the *narK* gene encoding a nitrite extrusion protein. These results may in part be responsible for the indirect effect of nitrate on *nar* expression. The NsrR transcriptional repressor also plays a role in nitrate respiration in *B. subtilis*. The most common target of this regulator in bacteria is the *hmp* gene, involved in NO detoxification, as observed in *B. subtilis* (Kommineni et al., 2012). However, it also regulates numerous other genes, including the nitrate reductase operon (*nasDEF*) (Figure 3; Nakano et al., 2006).

### Transcriptional Regulation of the Denitrification Pathway

The regulation of the denitrification pathway has been extensively studied in the γ-proteobacterium *P. aeruginosa* and in the α-proteobacterium *P. denitrificans*, and recently reviewed (Gaimster et al., 2018).

**FIGURE 4:**
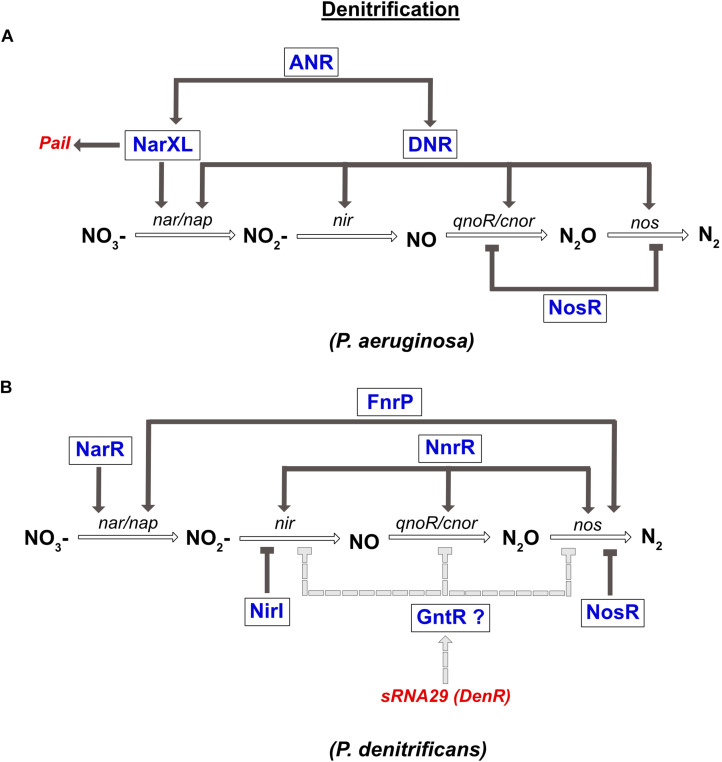
Regulation of denitrification in *P. aeruginosa* **(A)** and *P. denitrificans* **(B)**. **(A)** Expression of nitrate reductase gene (*nar*) is directly co-regulated by the NarXL TCS and the DNR transcriptional regulator. Both regulators (NarXL and DNR) are under the control of the FNR-like regulator ANR. DNR also activates expression of the nitrite reductase gene (*nir*). The nitric oxide reductase (*nor*) and the nitrous oxide reductase (*nos*) genes are repressed by the NosR transcriptional repressor. Transcription of the PaiI sRNA is controlled by the NarXL TCS. **(B)** Expression of nitrate reductase gene (*nar*) is co-regulated by NarR and FnrP. Expression of nitrite reductase (*nir*) and nitric oxide reductase (*nor*) genes are activated solely by NnrR. Expression of nitrous oxide reductase gene (*nos*) is co-regulated by FnrP and NnrR. NirI represses the expression of *nir*, while NosR repressed that of *nos*. Moreover, the recently identified DenR (also known as sRNA-29) inhibits *nir*, *nor* and *nos* expression, probably indirectly *via* a regulator of the GntR type.

In *P. aeruginosa*, the denitrification pathway is controlled by the TCS NarXL (sensing NO_3_^–^ and NO_2_^–^), two FNR like-proteins ANR and DNR, and the transcriptional regulator NosR, which is sensitive to NO and O_2_ (Figure 4). The *narK1K2GHJI* operon is activated by three transcriptional regulators, the ANR and DNR, and the NarXL TCS. In parallel, it was also shown that NarL represses expression of the *nap* operon during anaerobic growth (Van Alst et al., 2009). In the presence of NO, the expression of *nirS*, *norCB* and *nos* genes is activated by DNR (Arai et al., 1995, 2003). Moreover, ANR also controls the expression of *narXL* and *dnr* genes (Schreiber et al., 2007; Figure 4).

In *P. denitrificans*, three FNR-like proteins are involved in the regulation of denitrification: FnrP and NnrR that sense O_2_ and NO levels, and NarR that senses NO_3_^–^ and NO_2_^–^ (Lee et al., 2006; Bergaust et al., 2012; Crack et al., 2016; Figure 4). However, in contrast to FnrP, NnrR, and NarR lack the [4Fe-4S]^2+^ cluster. The NarR TF functionally replaces the NarXL TCS in α-proteobacteria, since no homologs have been identified so far (reviewed in Bueno et al., 2012). The *narGHIJ* operon is fully induced under anaerobic conditions *via* FnrP, and by nitrate *via* the NarR TF (Wood et al., 2001). The NnrR TF controls the expression of the *nir*, *nor*, and *nos* gene clusters (Figure 4; Saunders et al., 1999; Van Spanning et al., 1999; Bergaust et al., 2012). Finally, *nos* gene expression is also regulated by FnrP (Crack et al., 2016). In addition to these global regulators, two related proteins NirI and NosR, with metal ion-binding or thiol sites (CXXXCP motif), were specifically shown to regulate the expression of the *nir* and *nos* genes, respectively (Saunders et al., 1999). As mentioned above, the detrimental effect of NO is avoided by the interaction between nitric oxide reductase and nitrite reductase in *P. denitrificans* and *P. aeruginosa*. However, their co-regulation by NnrR is another way to control NO accumulation.

In the α-proteobacteria *B. japonicum*, *Rhodobacter capsulatus*, *Sinorhizobium meliloti*, and *R. sphaeroides*, similar redox sensitive TCSs have been identified, namely RegSR, RegBA, ActSR, and PrrBA, respectively. All these TCSs share a high sequence similarity in their C-terminal domain. They are involved in the regulation of different metabolic pathways, including nitrogen fixation, and can functionally replace each other (Emmerich et al., 2000). The FnrL regulator and the PrrBA TCS are important for photosynthetic gene expression during anaerobic growth of *R. sphaeroides*. PrrAB was also shown to induce expression of the nitrate reductase NirK, since a PrrA deletion decreases expression of the *nirK*-lacZ fusion (Laratta et al., 2002). Another key regulator in *R. sphaeroides* is the FNR-like protein NnrR, that senses NO, and that is also required for the expression of *nirK*, as well as that of the *norCBQD* operon encoding the nitric oxide reductase (Kwiatkowski and Shapleigh, 1996; Tosques et al., 1996; Laratta et al., 2002). This result shows that the NnrR regulon is indirectly controlled by the PrrAB TCS, since NO (synthetized by *nirK*) is necessary for NnrR function, and suggests that, in contrast to other denitrifying bacteria, *R. sphaeroides* uses nitrate respiration to ensure redox balancing during photosynthesis, rather than to support anaerobic growth.

In the ε-proteobacterium *W. succinogenes*, the *nap*, *nrf*, and *cnos* genes involved in nitrate respiration are upregulated in presence of nitrate, NO or N_2_O but not by nitrite or hydroxylamine (Kern and Simon, 2016). Three TFs, belonging to the Crp/FNR superfamily, are involved in the upregulation of these genes in specific growth condition, namely NssA, B, and C. Nss factors have an N-terminal effector domain and a C-terminal DNA binding domain. Further study will be necessary to understand how nitrate, NO and N_2_O are sensed by these different TFs. These TFs are homologous to NssR, previously identified in *C. jejuni* and involved in NO stress resistance (Elvers et al., 2005). However, in *C. jejuni*, NssR binding sites have not been identified upstream of the *nap* and *nrf* genes (Elvers et al., 2005).

In *S. coelicolor*, which possesses three nitrate reductases (Nar1, Nar2, and Nar3), the OsdRK TCS controls the expression of the *narGHJI2* operon (Fischer et al., 2019). Interestingly, Nar1 and Nar3 are not under the control of this TCS, but are induced under anaerobic conditions.

Finally, in the γ-proteobacterium *S. loihica* PV-4, the global regulator CRP1, which is widely present in bacteria, is necessary to be able to use nitrate in the DNRA pathway, while its paralog CRP2 is required for transcription of the nitrite reductase gene *nirK* for denitrification (Liu et al., 2020).

Other transcriptional regulators are involved in the control of genes linked to NO detoxification, such as NorR in *E. coli* which regulates *norV* and *norW* and have previously been reviewed by Spiro (2007). However, we consider these transcriptional regulators as beyond the scope of this review.

In addition to such complex transcriptional control of the respiration processes, it has more recently become evident that an additional layer of post-transcriptional regulation by non-coding RNAs is also crucial in the regulation of cellular respiration. sRNAs can rapidly evolve to adapt to specific conditions (reviewed by Dutcher and Raghavan, 2018) and, not surprisingly, recent discoveries have confirmed a role for sRNAs in nitrate respiration (see also Table 1).

**TABLE 1:** sRNAs that could play a role in nitrate respiration.

sRNA	TF	Target(s) related to nitrate respiration	References

DNRA pathway			
***Escherichia coli/Salmonella enterica***			

NarS	NarL*, FNR*	*nirC*	Wang et al., 2020

SdsN	RpoS (indirect?)	*narP*	Hao et al., 2016

RprA	Rcs phosphorelay	*narP*	Brosse et al., 2021

ArcZ	ArcA	*napF* (interaction detected by RIL-seq)	Melamed et al., 2016, 2020

DicF	? (accumulates under microaerobic conditions)	*narL* (direct?)	Melson and Kendall, 2019

FnrS	FNR*		Durand and Storz, 2010

RyhB	Fur	*nirB*, *narP* (likely direct) and *narL* (direct?)	Teixidó and Cortés, 2010; Wright et al., 2013; Hao et al., 2016

***Bacillus subtilis/Staphylococcus aureus***			

RoxS	ResDE*		Durand et al., 2015

**Denitrification pathway**			

***Paracoccus denitrificans***			

DenR	?	Transcriptional regulator of several denitrification genes (direct?)	Gaimster et al., 2019

***Pseudomonas aeruginosa***			

PaiI	NarL*, ANR* (via NarL?)		Tata et al., 2017

***Rhodobacter sphaeroides***			
PcrZ	PrrA*, FnrL* (indirect?)		Mank et al., 2012

*When known, the regulators controlling the sRNA synthesis (TF) and the targets relevant for nitrate respiration are indicated. TFs with an asterisk are known to be involved in the control of nitrate respiration. See text for details.*

## Small RNAs Involved in Nitrate Respiration

### sRNAs Involved in the DNRA Pathway

Small RNAs involved in the respiration on nitrate have been studied in greatest detail so far in the enterobacteria *E. coli* or *S. enterica*, where a wealth of data points to the involvement of several sRNAs in the DNRA pathway. As stated above, the NarX-NarL and NarQ-NarP TCSs play key roles in the regulation of the enzymes involved in this process, and a first link between sRNAs and nitrate respiration came from the identification of sRNAs that target these regulators.

SdsN_137_ is one of three sRNA isoforms and it represses expression of the *narP* response regulator gene by pairing to the translation initiation region of this mRNA (Hao et al., 2016). Interestingly, the two other confirmed targets of the SdsN sRNAs are also involved in nitrogen metabolism, as they encode NfsA, a major NADPH-dependent nitroreductase, and HmpA, a nitric oxide dioxygenase. In agreement with the role of NfsA in governing *E. coli* sensitivity to nitrofuran antibiotics, SdsN was found to protect cells against these compounds (Hao et al., 2016). The same study showed that the SdsN levels respond to the stress/stationary phase sigma factor RpoS, but also change in response to different nitrogen sources (e.g., ammonium or arginine), in a NarP-dependent manner. It is still unclear, however, whether RpoS and NarP are direct regulators of *sdsN* gene transcription.

More recently, RprA was identified as a second sRNA that directly controls *narP* mRNA expression through direct base-pairing with its translation initiation region. Of note, in addition to this canonical regulation, RprA also represses *narP* by acting at the very 5′ end of the *narP* mRNA, more than 100 nucleotides upstream of the *narP* start codon (Brosse et al., 2021). Even though the precise mechanism for this additional control is unclear, it is most likely to be indirect, suggesting the existence of yet other *narP* post-transcriptional regulators. Transcription of RprA sRNA is primarily controlled by the Rcs phosphorelay that responds to envelope stress (Majdalani et al., 2002), and previously reported targets of RprA include the gene encoding the RpoS global stress response protein, and others involved in biofilm formation, conjugation, acid resistance (Majdalani et al., 2001; Mika et al., 2012; Papenfort et al., 2015; Lalaouna et al., 2018), showing that this sRNA is not just dedicated to the control of the nitrate respiration.

Changing the levels of a regulatory protein may not always lead to an effect on its regulon (Batchelor and Goulian, 2003). This is especially true in the case of response regulators that most often need to be modified by phosphorylation to be active. At least in the case of *narP* control by RprA or SdsN_137_ sRNAs, the overproduction of either sRNA is sufficient to repress the expression of the *nap* operon, via their effect on *narP* regulation, suggesting that control by these sRNAs can really impact nitrate respiration (Brosse et al., 2021).

The *narL* mRNA, which encodes the response regulator of the NarX-NarL TCS, is also likely subjected to post-transcriptional control by the DicF sRNA. This sRNA accumulates in low oxygen conditions and its gene is present in one copy in the commensal *E. coli* K-12 genome, but in four copies in the enterohemorrhagic strain 0157:H7 (EHEC) (Murashko and Lin-Chao, 2017; Melson and Kendall, 2019). DicF regulates the expression of genes important for cell division and metabolism (Tetart and Bouché, 1992; Balasubramanian et al., 2016) and enhances EHEC virulence (Melson and Kendall, 2019). *narL* was among the mRNAs that were differentially expressed in RNAseq analysis performed in the EHEC strain in the presence and the absence of DicF. qPCR data further confirmed that *narL* mRNA levels increase in the strain deleted for the four *dicF* genes, and showed that overproduction of any one of the four DicF sRNAs reversed this effect. Whether this is due to a direct interaction between DicF and the *narL* mRNA, and whether this control is conserved in other bacteria are still open questions.

Finally, there are some indications that the RyhB sRNA could also play a role in the expression of *narP* and/or *narL* mRNAs. More precisely, RyhB overproduction significantly repressed the synthesis of a *narP-lacZ* translational fusion in *E. coli* (Hao et al., 2016), while qRT-PCR assays suggested that the two *Salmonella* RyhB homologs, also called RfrA and RfrB, can activate *narL* and repress *narP* expression (Teixidó and Cortés, 2010). Again, precise mechanisms for these controls remain to be determined, but a convincing pairing can be predicted between RyhB and the *narP* mRNA, suggesting a direct interaction in this case (Hao et al., 2016).

As stated above, many enzymes involved in nitrate respiration require metal cofactors such as iron and it is thus interesting that the iron-responsive RyhB sRNA may participate in the regulation of these pathways. This is further exemplified by the regulation of the genes encoding the NirBD nitrite reductase in enterobacteria, since *nirB*, the first gene of the *nirBDC*-*cysG* operon, was predicted as a RyhB target using the CopraRNA pairing prediction algorithm. This interaction was validated by compensatory mutations showing that RyhB represses *nirB* expression in *E. coli* (Wright et al., 2013). Conversely, however, the effects of the two RyhB homologs in *Salmonella* that have been proposed to activate *nirBDC* expression upon nitrosative stress may be indirect (Calderón et al., 2014).

In addition to sRNAs targeting TFs of the nitrate respiration pathway such as the NarP or NarL response regulators, several sRNAs whose transcription depends on these TFs were also identified. A first example is the NarS sRNA whose levels respond to FNR and NarL TFs (Wang et al., 2020). NarS has been recently identified as an sRNA derived from the 3′ UTR of the *narK* mRNA, encoding a nitrate and nitrite transporter, through processing by the essential endonuclease RNase E. Transcription of *narK* is under the direct control of FNR and NarL, which explains how these TFs also control the accumulation of the NarS sRNA. Interestingly, NarS is a Hfq-binding sRNA that represses the expression of the *nirC* gene, while leaving the expression of the other cistrons of the *nirBDC-cysG* operon unchanged (Wang et al., 2020). As NirC is involved in nitrite uptake, it was proposed that the negative control of *nirC* by NarS could limit nitrite re-import under conditions of *narS* expression, where nitrite can be exported *via* NarK or reduced to ammonium.

Other cases of sRNAs whose transcription is under the control of TFs of the nitrate respiration pathway have been reported. As previously mentioned, FNR is a major regulator of gene expression in response to anaerobiosis. It regulates hundreds of genes, including genes whose products are directly related to nitrate respiration such as the *nap*, *nir*, and *nrf* operons in *E. coli* (see above). The FnrS sRNA was recognized early on as part of the FNR regulon (Durand and Storz, 2010) and is thus expressed under anaerobic conditions. Of note, *fnrS* transcription is also controlled by two other major TFs, ArcA (positively) and CRP (negatively), which allows further accumulation of FnrS, under anaerobic conditions and in glucose-based media, respectively (Durand and Storz, 2010). More than 30 mRNAs are repressed following FnrS pulse-overproduction; they encode proteins involved in diverse functions, such as central metabolism, energy metabolism, aerobic respiration, oxidative stress, that are dispensable under anaerobiosis. FnrS-targets include the NfsA nitroreductase, whose mRNA is also targeted by SdsN, and the CydDC glutathione transporter, important for periplasmic redox control. Interestingly, expression of the *cydDC* operon is additionally controlled at the transcriptional level by ArcA, NarL, and FNR, with these two latter regulators allowing induction of expression under anaerobic conditions in the presence of alternative electron acceptors, such as nitrate or nitrite (Cook et al., 1997).

Importantly, the ArcA response regulator not only contributes to FnrS synthesis, but also directly represses the transcription of ArcZ, another Hfq-dependent sRNA that targets multiple mRNAs, including *rpoS*, *eptB*, *sdaC*, *tpx*, *mutS*, or *tolC* (Papenfort et al., 2009; Mandin and Gottesman, 2010). The ArcB-ArcA TCS is activated under microaerobic conditions (Alexeeva et al., 2000) and its regulon includes many genes involved in aerobic respiration, thereby partially overlapping the FNR regulon. In addition, and in contrast to FNR, ArcB-ArcA does not directly control expression of genes of the nitrate respiration pathway. However, recent data from RNA–RNA interactome studies strongly support an interaction between the ArcZ sRNA and the *napF* mRNA and hence suggest an indirect role for ArcA in *napF* expression through the ArcZ sRNA (Melamed et al., 2016, 2020).

Transcriptional control of sRNAs by TFs involved in nitrate respiration also is true in Gram-positive bacteria. Transcription of RoxS, the only base-pairing sRNA that is conserved in *B. subtilis* and *Staphylococcus aureus* (where it is known as RsaE), is for instance directly controlled by the ResDE TCS (or its homolog SsrAB in *S. aureus*), allowing RoxS induction in the presence of NO (Durand et al., 2015). RoxS has many targets, several of which are involved in carbon and energy metabolism, or in amino acid transport. However, it is not yet clear whether this sRNA plays a direct role in nitrate respiration.

### sRNAs Involved in the Denitrification Pathway

Although the biology of sRNAs in general has been less studied in denitrifying bacteria, it is already clear that sRNAs are involved in controlling denitrification. A recent transcriptome analysis determined the sRNA composition of *P. denitrificans*, the model bacterium for the study of denitrification, under different denitrifying conditions: N_2_O-producing or N_2_O-consuming during anaerobiosis, versus aerobic growth without N_2_O (Gaimster et al., 2016). These conditions were set-up by adding CuSO_4_ to the medium (N_2_O-consuming) or omitting it (N_2_O-producing), as expression of the nitrous oxide reductase NosZ, itself a copper-dependent metalloenzyme required for the last step of the denitrification pathway, depends on copper (Sullivan et al., 2013). This study allowed the identification of several sRNAs with a potential role in denitrification. Among these, the DenR sRNA (for denitrification repressor, previously known as sRNA-29) was shown later to impact denitrification by repressing the expression of several genes involved in this process, such as *nirC*, *norBC*, or *nosZ*, encoding nitrite reductase, subunits of nitric oxide reductase, and nitrous oxide reductase, respectively. These controls are likely indirect and mediated by DenR promoting the synthesis of a GntR-type transcriptional regulator, whose expression follows that of the sRNA, i.e., is highest in the absence of N_2_O and decreases as N_2_O levels increase (Gaimster et al., 2019). Of note, this transcriptional regulator was not previously known to control denitrification. As a result, DenR overproduction stalls denitrification at the nitrite reduction step, thereby increasing nitrite levels, while limiting production of NO and N_2_O.

In *P. aeruginosa*, another denitrifying bacterium, the 126-nt PaiI sRNA (for *P. aeruginosa* anaerobically induced RNA I) was identified by RNAseq as the most enriched sRNA under anoxic conditions. Further work showed that PaiI accumulates under anaerobic conditions in the presence of nitrate in an ANR- and NarXL-dependent manner (Tata et al., 2017). The presence of a close-to-consensus NarL site in the PaiI promoter sequence additionally suggests that NarL directly controls *paiI* transcription, while the ANR-regulation is most likely indirect *via* activation of NarXL by ANR (Figure 3). Interestingly, PaiI-deleted cells display defects in survival after a shift to anoxic conditions in the presence of nitrate and the activity of nitrite reductase is decreased in the absence of the sRNA (Tata et al., 2017). The molecular basis of these observations is still unclear, but these data nonetheless clearly point to a role for the PaiI sRNA in denitrification.

The PcrZ sRNA of the α-proteobacterium *R. sphaeroides* is another example of an sRNA responding to regulators of denitrification genes. PcrZ synthesis is induced under low oxygen conditions and this is dependent on the PrrA response regulator and the FnrL TF (Mank et al., 2012). While the presence of a consensus site for PrrA in the *pcrZ* promoter strongly suggests a direct regulation, this may not be the case for FnrL control (Mank et al., 2012). Both PrrA and FnrL are key regulators of *R. sphaeroides* anaerobiosis gene expression, and their regulons include genes for the photosynthesis apparatus. In turn, the PcrZ sRNA represses expression of several photosynthesis genes through direct pairing with their mRNAs. Interestingly, transcription of these mRNAs is itself activated by PrrA and FnrL; PcrZ thereby participates in an incoherent feed-forward loop to regulate the expression of photosynthesis genes (Mank et al., 2012). PcrZ does not appear to be directly involved in denitrification at this stage, but is more likely to play a fine-tuning role in the metabolic versatility of *R. sphaeroides*.

It was also recently proposed that an antisense RNA is produced from the intergenic region between the *norQ* and *norD* genes of the *norECBQD* operon, encoding an NO reductase in the facultative denitrifying bacterium *Agrobacterium fabrum* (Lecomte et al., 2020). A possible role for this antisense RNA could be to promote denitrification, since N_2_O production is reduced more than three-fold after inactivation of this antisense RNA. However, further studies are required to both confirm the existence of this regulatory RNA and address its precise mode of action.

## Conclusion

Nitrate respiration is an important metabolic pathway, especially when oxygen is limiting, and is regulated at multiple levels. An important control of the nitrate respiration process is exerted at the transcriptional level allowing the integration of numerous environmental signals to precisely control the expression of enzymes involved in this pathway. More recent studies have shown the additional importance of post-transcriptional regulation: the involvement of sRNAs in nitrate respiration is beginning to be recognized, even though this is so far restricted to only a few model bacterial species. In addition, even in these species, their role is very likely still underestimated. Many mRNAs encoding either enzymes or TFs of the DNRA pathway were for instance significantly enriched upon Hfq co-immunoprecipitation in several studies in enterobacteria, suggesting that they could be subject to post-transcriptional control by base-pairing sRNAs as well. Furthermore, high-throughput analyses relying on the ligation of interacting RNAs following their co-immunoprecipitation with Hfq or ProQ, identified many more putative sRNAs interactions with mRNAs whose products are related to the DNRA pathway (e.g., several *nap*, *nar*, *nrf*, or *nir* genes) (Melamed et al., 2016, 2020). In addition, in *S. meliloti*, Hfq was found to directly interact both upstream and within the *fixLJ* ORF, encoding an O_2__–_sensing TCS, and to play a role in its expression (Gao et al., 2016). This control is expected to both directly and indirectly modulate the expression of FixLJ targets, including the *nif* operon (*nifHDK*). Although it is still unknown whether *fixLJ* regulation by Hfq involves an sRNA or not, this is yet another illustration that the post-transcriptional control of nitrate respiration genes is far from being fully understood.

Finally, it is important to emphasize that, at least in the non-denitrifying bacteria, most studies of gene regulation by sRNAs are performed under aerobic conditions, where expression of genes for nitrate respiration may be much lower than under anaerobic conditions. There is no doubt that additional work under more favorable conditions for nitrate respiration would unravel novel sRNA regulations.

Overall, it thus appears that, as for other important metabolic pathways, nitrate respiration, and more generally nitrogen metabolism, are precisely controlled by multiple regulators acting both at the transcriptional and post-transcriptional levels. In addition to these controls of gene expression, regulation of nitrate respiration could be achieved by modulating the activity of enzymes. Indeed, it was shown that the activity of the *E. coli* NarGHI nitrate reductase was controlled by the dynamics of the complex, that assembles to the cell poles under anaerobic conditions (Alberge et al., 2015). As more details of these regulatory mechanisms are revealed, ideally in a large number of bacterial species, it will be interesting to determine the relative contributions of the diverse regulators involved and their respective advantages in relation to the different nitrate respiration pathways and the environmental conditions encountered by bacteria.

## Author Contributions

Both authors listed have made a substantial, direct and intellectual contribution to the work, and approved it for publication.

## Conflict of Interest

The authors declare that the research was conducted in the absence of any commercial or financial relationships that could be construed as a potential conflict of interest.
